# Right posterior diaphragmatic hernia (Bochdalek) with liver involvement and alteration of hepatic outflow in adult: a case report

**DOI:** 10.1186/s40064-016-3221-2

**Published:** 2016-09-14

**Authors:** Filippo Banchini, Roberta Santoni, Antonio Banchini, Flavio Cesare Bodini, Patrizio Capelli

**Affiliations:** 1 U.O. Chirurgia Generale Vascolare Senologica, Ospedale Guglielmo da Saliceto, Via Taverna 49, cap, 29100 Piacenza, Italy; 2 Unità di Medicina Legale, Dipartimento di Scienze Biomediche, Biotecnologiche e Traslazionali – S.Bi.Bi.T., Università degli Studi di Parma, Parma, Italy; 3 U.O. Radiologia, Ospedale Guglielmo da Saliceto, Piacenza, Italy

**Keywords:** Liver herniation, Right diaphragmatic hernia, Bochdalek, Hepatic vein

## Abstract

**Introduction:**

Posterior right diaphragmatic hernia is rare in newborn patients but when present, is accompanied by high mortality. Pulmonary hypoplasia seems to be the main cause of death but the presence of liver involvement remains one of the reasons for poor prognosis even when intrauterine surgery is performed.

**Case Description:**

In this article, we will present a rare case that was diagnosed by chance in a 65-year old adult presenting with an adenocarcinoma of the rectosigmoid junction and a right Bochdalek hernia with liver herniation and modification of the hepatic vein outflow with a natural right to left shunt.

**Discussion:**

Diaphragmatic repair was performed on the patient with a mash and simultaneous colorectal resection. Intraoperatively, the exceptional natural modification of the hepatic outflow and alteration of the caval system was evident.

**Conclusion:**

This case report represent an extremely rare anatomic variation and could be useful to give new important information on the evolution that occur in foetal life.

## Background

Congenital diaphragmatic hernia (CDH) is a malformation that occurs mainly during foetal life causing abnormal pulmonary development with subsequent pulmonary hypoplasia and hypertension. In approximately 85 % of cases, it occurs on the left side, whereas involvement of the right side, “liver up”, occurs only in 8 % of cases. Foetal surgery correcting the defect was studied in the 1990s with promising results, but this approach then was abandoned due to a lack of increased survival over standard postnatal care. Currently, exclusively tracheal occlusion is performed in selected cases to prevent pulmonary hypoplasia. Despite significant progress in neonatal intensive care and the use of extra-corporeal membrane oxygenation (ECMO) that has improved survival and reduced indication for foetal surgery, mortality still remains very high.

## Case report

**Fig. 1:**
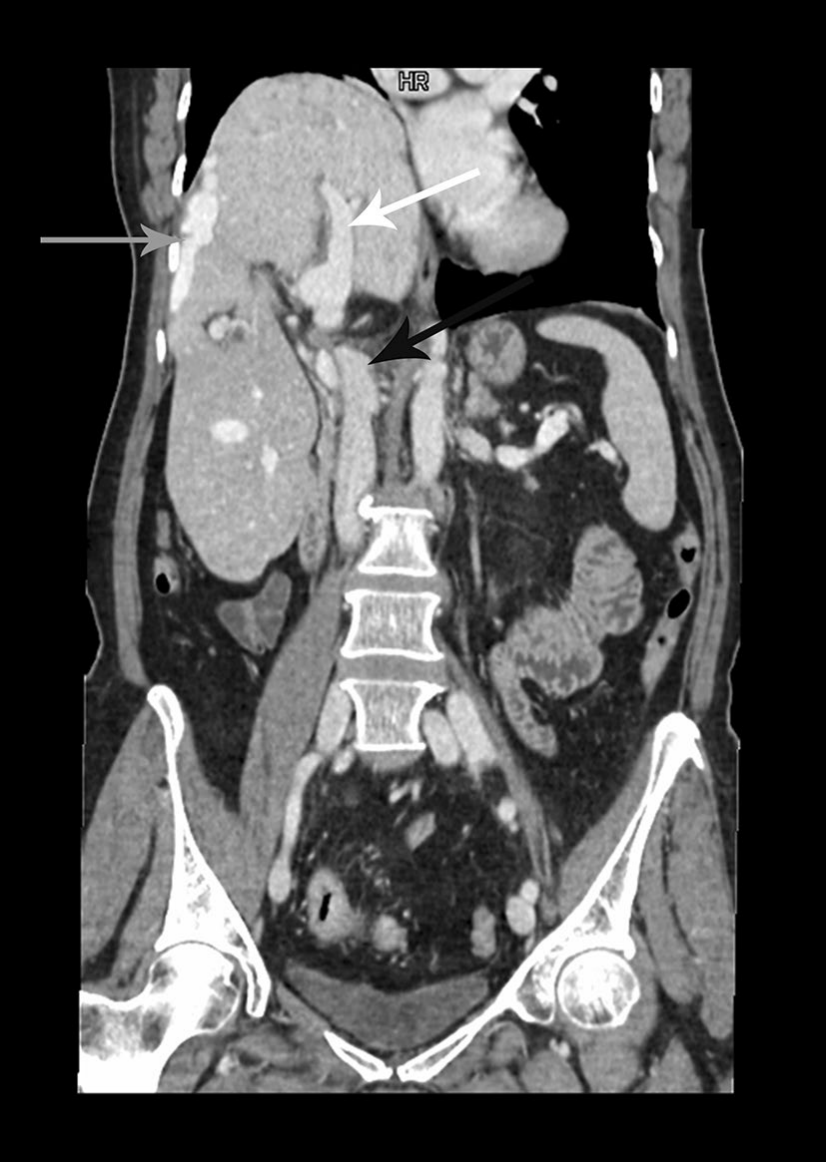
*White arrow* right portal branch, *black arrow* inferior vena cava, *gray arrow* hepatic shunt

**Fig. 2:**
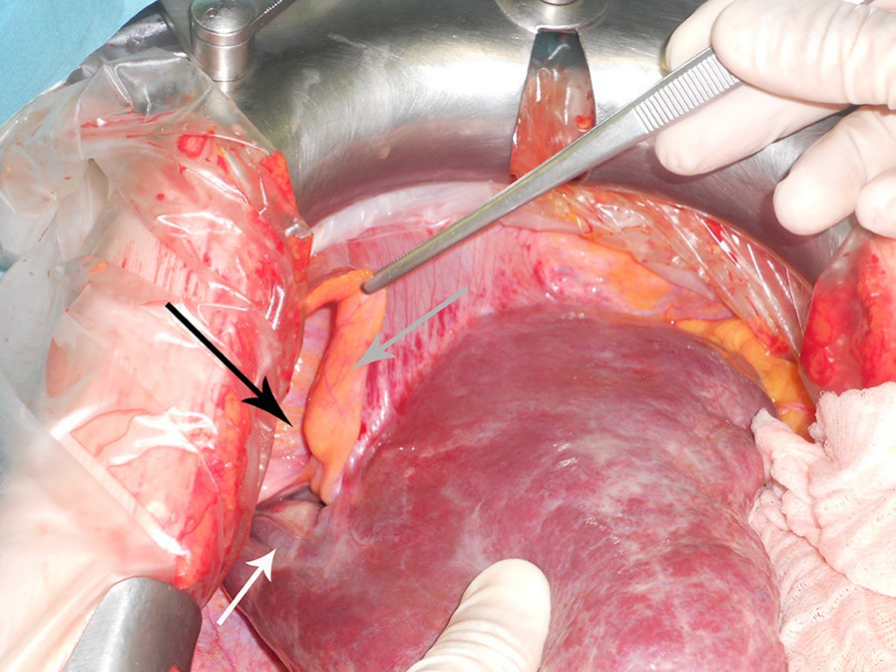
*White arrow* hepatic shunt, *black arrow* diaphragm, *gray arrow* round ligament

**Fig. 3:**
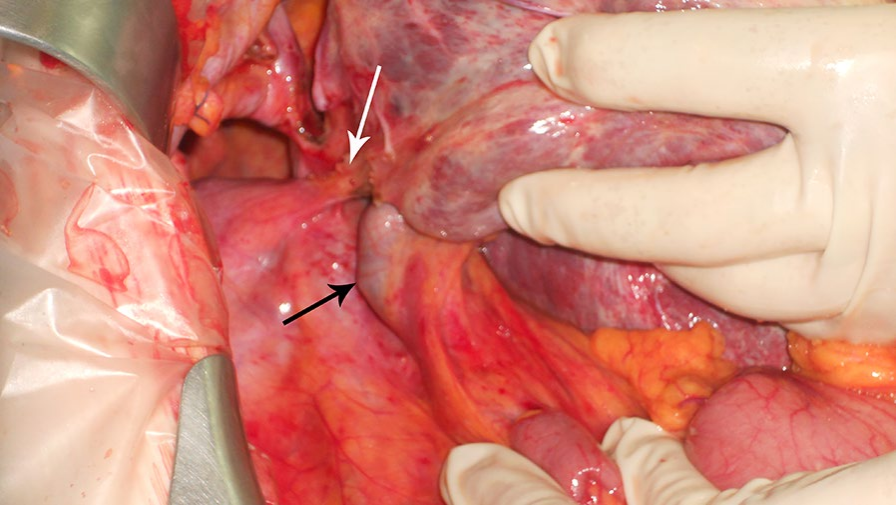
*White arrow* ligament compressing the inferior vena cava, *black arrow* inferior vena cava above the liver

**Fig. 4:**
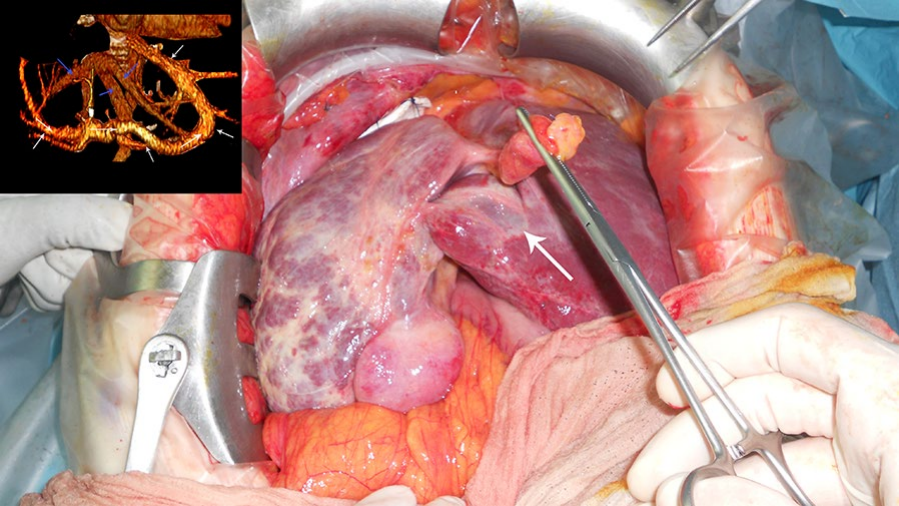
*White arrow* hepatic veins and shunt in front of the round ligament *blue arrow* portal system

A 65-year old woman was hospitalized after reporting abdominal pain and faecal impaction. Blood samples were normal and a chest X-ray revealed hypoplasia of the right lung with severe thoracic scoliosis. A colonoscopy and gastrografin enema were performed with diagnosis of adenocarcinoma of the rectosigmoid junction. Pre-operative staging was performed with a CT scan that highlighted a right diaphragmatic hernia with involvement of the right kidney and subsequent migration of the right liver into the thorax. This herniation led to torsion of the caval axis and occlusion of the right and middle hepatic veins with modification of liver outflow, thus creating a right to left hepatic vein shunt passing anteriorly to the umbilical recess (Fig. 1). An alteration of the retrohepatic cava vein was also demonstrated with suspected double caval system. The right lung presented absence of the inferior segment with bronchial ending without atelectasis. The patient underwent combined hernia repair and rectosigmoid resection. Intraoperative findings revealed a large right posterior diaphragmatic hernia without a sac opened in the thorax. Exclusively the left lobe remained inside the abdominal cavity with traction of the hepatic hilum under the right diaphragm with the round and falciform ligament overlying the diaphragm sustaining the liver (Fig. 2). The liver was rotated in the abdominal cavity intraoperatively: this retraction revealed a ligament between the diaphragm and the liver determining compression of the inferior vena cava (Fig. 3). The ligament was therefore, dissected in order to restore the vena cava’s normal diameter. Also the right kidney was reduced in the abdominal cavity and hernia repair was performed with dual mash sutured with non-absorbable separated stitches to the diaphragm. A shift of the liver outflow was seen with a natural by-pass inverting flow from the right and middle hepatic veins to a large accessory hepatic vein in the parenchymal bridge in front of the umbilical recess and then to the left hepatic vein (Fig. 4). Sigmoid resection with mechanical colorectal anastomosis was performed at the same time. The postoperative course was uneventful and the patient was discharged eight days after surgery.

## Discussion

Abnormal pleuroperitoneal canal closure is the cause of diaphragmatic hernias at 8 weeks of gestation. Between 50 and 90 % of cases are diagnosed prenatally via ultrasonography (Nakayama et al. 1985; Adzick et al. 1989). When they occur in the posterior side, they are called Bochdalek hernia, when in the anterior side, they are called Morgagni hernia. In rural areas, mortality rate caused by pulmonary hypoplasia remains high (Cigdem et al. 2007). 77 % die despite optimal pre-and post-natal care (Harrison et al. 1990). Incidence is 1 in 2000–5000 live births and more than 85 % of cases occur on the left side (Shenoy and Johri 2013). However, a recent case series presented by Cigdem et al. (2007) showed a very low incidence, 6 % and three out of the eighteen cases occurred on the right side.

Symptoms are more often present in children with pulmonary distress, whereas in adults, chronic dyspnoea and chest pain are occasionally present. Moreover, intestinal problems caused by strangulated hernia (Kocakusak et al. 2005; Kanazawa et al. 2002; Rout et al. 2007) can appear.

In about 10–38 % of cases, a sac is present that can rupture in adult life with the appearance of acute symptoms or strangulation (Kocakusak et al. 2005). Correction of congenital diaphragmatic hernia is possible in utero. However, liver involvement leads to significantly high mortality (Harrison et al. 1997). Currently, however, in utero surgery should be considered exclusively experimental and for optimal lung development, tracheal occlusion is required and should be performed as early as possible between 26 and 28 weeks of gestation. Experimental studies carried out on in utero CHD liver-up showed a decreased chance of survival related to technical problems in reducing the incarcerated liver in the abdominal cavity, jeopardising umbilical venous return (Harrison et al. 1990). With regards to technical problems, our experience could be useful to show what problems could arise due to the liver occupying the thorax and following surgery, when the liver is reduced in the abdomen. During foetal life, blood circulation passes through the umbilical vein to the Rex umbilical fissure, then to the Arantius ligamentum venosum and left hepatic vein and cava. In our case, the occlusion at the insertion of the right and middle hepatic veins, thanks to the persistence of the left hepatic vein flow probably maintained by foetal circulation, allowed the formation of a natural intrahepatic shunt from the right and middle hepatic veins to the left hepatic vein and also the presence of caval stenosis allowed formation of paracaval azygos and hemiazygos hypertrophy. The absence of this natural shunt could explain the high rate of mortality due to diaphragmatic hernia in foetal life when the liver is involved, but also in perinatal life during which occlusion of the umbilical vein and Arantius ligamentum could cause retracting fibrosis at its insertion in the left hepatic vein with consequent occlusion or stenosis. Moreover, the high rate of mortality in the operated foetus with liver involvement could be explained in the rotation manoeuvre of the liver in the abdomen. In fact, this could cause a caval stenosis with two possible consequences: complete caval occlusion under the origin of hepatic vein and interruption of caval flow that could be compensated by persistence of the partial caval lumen or extracaval venous system (azygos and hemiazygos) if present or direct occlusion at the origin of one or more hepatic veins with consequent liver congestion and also occlusion of the transhepatic umbilical flow at the insertion of the Arantius ligamentum venosum at the left hepatic vein.

For this reason during surgery, rotation of the liver must be performed with great caution checking whether a stenosing ligament is present strangulating the vena cava, thus dissecting the ligament. To our knowledge, no similar cases have ever been reported in the literature.

## Conclusion

 Diaphragmatic hernias are rare and liver involvement remains a poor prognostic factor. The objective of this extraordinary case report was to try and give new important information on the evolution and natural modifications that occur in foetal life in patients affected by right diaphragmatic hernia with liver involvement, hoping this could be useful in the future.
